# Unexpected rabies variant identified in kinkajou (*Potos flavus*), Mato Grosso, Brazil

**DOI:** 10.1080/22221751.2020.1759380

**Published:** 2020-05-14

**Authors:** Paulo Ricardo Dell’Armelina Rocha, Andres Velasco-Villa, Ernani Machado de Lima, Angela Salomoni, Alice Fusaro, Eunice da Conceição Souza, Risia Lopes Negreiros, Vera Lúcia Zafino, Gianpiero Zamperin, Stefania Leopardi, Isabella Monne, Paola De Benedictis

**Affiliations:** a Graduate Program in Environmental and Experimental Pathology, Paulista University, São Paulo, Brazil; b Graduate Program in Veterinary Sciences, Faculty of Veterinary Medicine, University of Turin, Turin (TO), Italy; c Poxvirus and Rabies Branch, Division of High-Consequence Pathogens and Pathology, National Center for Emerging and Zoonotic Infectious Diseases, CDC, Atlanta, GA, USA; d Laboratorio de Apoio a Saude Animal (LASA), Complexo do Instituto de Defesa Agropecuaria do Estado de Mato Grosso, Cuiaba, Brazil; e FAO and National Reference Centre for rabies, Istituto Zooprofilattico Sperimentale delle Venezie, Legnaro (PD), Italy

**Keywords:** Rabies, Brazil, *Cebus apella*, *Potos flavus*, Chiroptera, public health

## Abstract

A second case of a novel rabies variant described once in a capuchin monkey from Mato Grosso, Brazil, was discovered in a rabid wild kinkajou from the same region, indicating a public health risk following exposure to either of the two animals.

Rabies is a viral fatal encephalomyelitis caused by all members of the *Lyssavirus* genus. It affects humans mostly through bites of rabid animals. Over its evolutionary history, rabies virus (RABV), which is the prototype species of the genus, has established independent transmission cycles in mammals (of bat and dog origin) – primarily through host shifts – overcoming species, geographic and ecological barriers [1,2]. In Brazil, RABV has established independent cycles that eventually have become endemic in wild canids (i.e. the crab eating and the hoary foxes *Cerdocyon thous*, *Pseudalopex vetulus*, and *Dusicyon vetulus*), in one nonhuman primate (NHP) (the marmoset monkey *Callithrix jacchus*), in the vampire bat (*Desmodus rotundus*) and in several species of insectivorous bats [3–5].

Here we describe the genetic characterization of a RABV variant from Cuiaba, Mato Grosso state, Brazil, whose host was retrospectively identified as a wild kinkajou (also known as Jupará or honey bear, *Potos flavus*) (Appendix).

The RABV variant obtained from this rabid Brazilian kinkajou (BRkj) represents the second case of a novel strain previously described in a rabid tufted capuchin monkey, *Cebus apella* (also referred as BRmk1358) from Marcelândia, Mato Grosso, which was determined to pertain to the RABV bat clade by phylogenetic analysis [6].

BRkj was found in a sample repository of 183 specimens collected from a rabies epizootic in bovines and equines that had occurred in Mato Grosso in the period of 2007–2011 (Appendix, Figure 1 and the Appendix Table depict information on each sample). All brain samples from this repository were genetically typed by sequencing of partial N gene amplicons (Appendix). Partial (603 nt) N gene sequences were aligned with RABV variants so far reported in the Americas. According to maximum likelihood (ML) reconstructions, all RABVs collected from bovines (*n *= 162) and equines (*n *= 20) grouped within four of the previously described Latin American *Desmodus rotudus* genetic groups, namely Ib, Id, IVb and VIb [7] (Appendix Figure 1). Most of the characterized sequences belonged to the group VIb (*n *= 140) and to a lesser extent Ib ( = 21), both of them widely circulating in Latin America (Argentina, Uruguay, Peru and Brazil) and in the different areas of Mato Grosso state. On the other hand, individuals sampled from the easternmost areas of Mato Grosso, bordering Goias and Tocantins states (*n *= 21), harboured RABVs of groups IVd and Id (one sequence). So far, these two genetic groups were identified in Brazil only. Out of the 183 samples under investigation, BRkj sequence was the only one separated from the *Desmodus rotondus* lineages and segregated within the bat clade together with the BRmk1358 strain (GenBank accession number AB810256) [6]. Complete genome sequencing (11,836 bp) of BRkj by a metagenomics approach confirmed a 99.99% nucleotide sequence identity with the BRmk1358 strain [6], corroborating that these two isolates are essentially the same RABV variant (Figure 1). All sequences were submitted to GenBank under accessions nos. MK910399-MK910580, MK920923 and MK990569, from viral and host genome sequences, respectively. Moreover, data reporting the entire sequences from the Kinkajou (*Potos flavus*) sample were deposited at the Brazilian System of the National Genetic Patrimony and The Associated common traditional knowledge (https://sisgen.gov.br/paginas/login.aspx), under accession number A4771EF.

**Figure 1.**
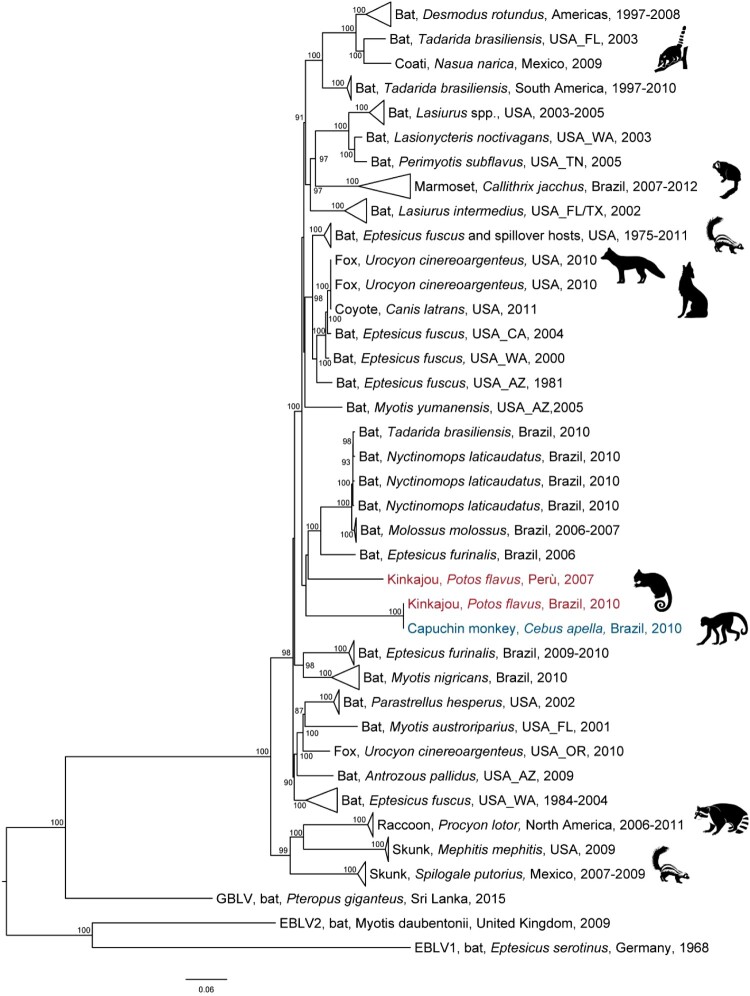
Legend. Maximum-likelihood phylogenetic tree of 92 complete genomes of bat associated rabies variants circulating in the Americas. For each variant, the following information is provided: common and scientific names of the host, country and year(s) of detection. The animal icon at the right of the branch indicates bat to terrestrial spills-over and eventual host-species jump. The rabies variants found in kinkajou (*P. flavus*) and capuchin monkey (*C. apella*) are indicated in red and blue, respectively.

Maximum likelihood phylogenetic reconstructions using whole genome sequences demonstrated that BRkj and BRmk1358 grouped consistently within the bat clade, but clearly segregated out of all extant RABVs associated with bats, forming a paraphyletic group similarly to another RABV variant found in the kinkajou in Peru (Figure 1). This data suggests that both Brazilian and Peruvian kinkajou variants likely have their evolutionary origin in bats. The establishment of new RABV variants in terrestrial mammals following host shift from bat hosts is more clearly supported for other RABV emergent clades, such as in the marmoset, in the Mexican coati, in the gray fox as well as in the Flagstaff Arizona skunk, which have a more recent close relative in extant bat-associated RABV variants (Figure 1) [1,2] . In our case, the average nucleotide identity (ANI) analysis conducted across the N gene and the complete genome sequences showed marked divergence of these variants compared with extant bat-associated ones (ANI matrix available on request). In particular, the most closely related bat-associated RABV variants to BRkj were identified in *Eptesicus fuscus* and *Myotis yumanensis* from North America with 87%, 86% and 86%, 85% for complete N genes and complete genomes, respectively. These data suggest that BRkj/BRmk1358 is unlikely to have emerged from a recent host shift from known bat-associated RABV variants, being rather established in mesocarnivores, similarly as it has been suggested for the North American raccoon and skunk variants located at the most ancestral branches in the tree (Figure 1) [1,2]. Nevertheless, due to single findings of this variant in two different hosts and considering the vast biodiversity present in Mato Grosso state, the hypothesis of the kinkajou and the tufted capuchin monkey representing spillover hosts of a cryptic cycle not yet identified in a bat could not be fully discarded.

ANI (Average Nucleotide Identity) values between BRkj and the Peruvian Kinkajou variant were 85% for complete N gene and 84% in all concatenated cistrons for the whole genome, supporting that BRkj/BRmk1358 and the variant found in kinkajous in Peru had been independently introduced in this species likely from bats. Similarly, this new variant was not related to any strain reported in NHPs, including marmoset. Although NHPs such as marmoset, capuchin, macaque and squirrel monkeys, as well as chimpanzees, have been associated with events of human rabies exposure in several countries across Africa, Asia and the Americas, there is a severe lack of rabies laboratory confirmation and characterization in presumptively rabid NHPs and consequently a lack of information on the RABV variants affecting them [8]. Thus, there is not consistent evidence to suggest that any NHPs other than marmosets may be able to sustain an independent rabies cycle or maintain their own RABV variant. The rabid tufted capuchin monkey reported in Brazil seems to be the first case genetically typed in contemporary times and no further cases of this variant in this species have been reported ever since [6]. A few investigations have displayed the capability of the genus *Cebus* in developing RABV neutralizing antibodies (rVNA) [9,10]. Altogether, virological and serological findings suggest that capuchin monkeys in the Amazonian region have been exposed to RABV, as for other likely exposed wildlife and for humans in the Peruvian Amazon [11,12], but do not prove any implication of *Cebus apella* as a primary host of the infection. On the other hand, kinkajous within the Peruvian Amazon have been affected for more than 5 years by a distinctive variant not yet detected in bats [13]. Although there is significant genetic distance between the Peruvian kinkajou variant and BRkj, its steady circulation in kinkajous may argue this species would be more prone to sustain a rabies cycle than tufted capuchin monkeys. Of note, based on phylogenetic analysis of the partial cytochrome B sequence, we were able to identify BRkj host as belonging to the subclade 5b (Appendix Figure 2), previously known to be mainly based in the Atlantic Forest. This and previous findings suggest that geographical barriers have likely played an important role in isolating kinkajous and their pathogens in Mato Grosso state, Brazil, from those in the Amazon Forest, mostly spread in Peru and Bolivia as well [14].

Interestingly, the tufted capuchin monkey shares its home range with the more broadly distributed kinkajou. Both species are frugivorous so that aggressive behaviour might be expected over food resources, especially during the dry season when fruit is scarcer [15]. Albeit ecological niches of these animals are predicted not to overlap, *C. apella* being mostly active during the day while *P. flavus* is a strict nocturnal species, abnormal behaviour when they become rabid might play a key role for disease spillover. On the other hand, an unrevealed bat host might be responsible for the natural maintenance of such a RABV variant. Indeed, possible interactions between bats and kinkajou might be related with accidental encroachment during the nighttime, while capuchin monkeys might include small bats in their diet [14,15]. Of note, despite being strictly arboreal and having a limited adaptation to urban environments and captivity, kinkajous and capuchin monkeys are hunted for pet trade, which increases the chance for human exposure. Thereby, rabies post exposure prophylaxis is recommended following human exposure in the wild or while in captivity, particularly when animals could not be quarantined for observation or brain tissues were not adequate or available for laboratory rabies diagnosis.

## Supplementary Material

Supplemental Material
