# Supplementary material for: Unexpected rabies variant identified in kinkajou (Potos flavus), Mato Grosso, Brazil
Source: Emerg Microbes Infect. 2020 May 14;9(1):851–4. doi: 10.1080/22221751.2020.1759380 (PMC7269027; doi:10.1080/22221751.2020.1759380)
Supplement: Supplemental Material [file TEMI_A_1759380_SM7515.zip › 1759380/Appendix Table 1.docx]

| **Sample ID** | **GenBank accession number** | **Host** | **Year** | **Location** |
| --- | --- | --- | --- | --- |
| BRbv163-07 | MK910502 | bovine | 2007 | Poxoreu |
| BRbv633-07 | MK910507 | bovine | 2007 | NovoSaoJoaquim |
| BRbv610-07 | MK910500 | bovine | 2007 | Poxoreu |
| BRbv01-08 | MK910436 | bovine | 2008 | RosarioOeste |
| BRbv06-08 | MK910515 | bovine | 2008 | Guiratinga |
| BRbv08-08 | MK910402 | bovine | 2008 | RosarioOeste |
| BRbv26-08 | MK910487 | bovine | 2008 | NovaOlimpia |
| BRbv31-08 | MK910501 | bovine | 2008 | Poxoreu |
| BRbv45-08 | MK910517 | bovine | 2008 | Guiratinga |
| BRbv67-08 | MK910437 | bovine | 2008 | Acorizal |
| BRbv80-08 | MK910516 | bovine | 2008 | PedraPreta |
| BRbv81-08 | MK910468 | bovine | 2008 | Araputanga |
| BRbv82-08 | MK910534 | bovine | 2008 | Cuiaba |
| BRbv92-08 | MK910451 | bovine | 2008 | BarraDoBugres |
| BRbv93-08 | MK910467 | bovine | 2008 | RosarioOeste |
| BRbv103-08 | MK910479 | bovine | 2008 | Araputanga |
| BRbv122-08 | MK910464 | bovine | 2008 | SJosequineuatroMarcos |
| BRbv123-08 | MK910401 | bovine | 2008 | Araputanga |
| BRbv124-08 | MK910478 | bovine | 2008 | Araputanga |
| BRbv140-08 | MK910443 | bovine | 2008 | Acorizal |
| BRbv148-08 | MK910504 | bovine | 2008 | Poxoreu |
| BRbv191-08 | MK910444 | bovine | 2008 | RosarioOeste |
| BRbv209-08 | MK910514 | bovine | 2008 | SaoJoseDoPovo |
| BRbv217-08 | MK910519 | bovine | 2008 | Rondonopolis |
| BRbv218-08 | MK910400 | bovine | 2008 | Araputanga |
| BRbv221-08 | MK910477 | bovine | 2008 | Araputanga |
| BRbv240-08 | MK910446 | bovine | 2008 | RosarioOeste |
| BRbv417-08 | MK910499 | bovine | 2008 | Poxoreu |
| BRbv427-08 | MK910536 | bovine | 2008 | RosarioOeste |
| BRbv475-08 | MK910449 | bovine | 2008 | Denise |
| BRbv21-09 | MK910432 | bovine | 2009 | SaoJoseDosQuatroMarcos |
| BRbv41-09 | MK910445 | bovine | 2009 | Nobres |
| BRbv43-09 | MK910533 | bovine | 2009 | Cuaiaba |
| BRbv101-09 | MK910535 | bovine | 2009 | NovaMutum |
| BRbv130-09 | MK910404 | bovine | 2009 | Jauru |
| BRbv139-09 | MK910490 | bovine | 2009 | MirassolD'Oeste |
| BRbv143-09 | MK910488 | bovine | 2009 | NovaMarilandia |
| BRbv189-09 | MK910491 | bovine | 2009 | TangaraDaSerra |
| BRbv226-09 | MK910439 | bovine | 2009 | Acorizal |
| BRbv254-09 | MK910469 | bovine | 2009 | Araputanga |
| BRbv257-09 | MK910532 | bovine | 2009 | NovoSaoJoaquim |
| BRbv259-09 | MK910513 | bovine | 2009 | PedraPreta |
| BRbv260-09 | MK910522 | bovine | 2009 | PedraPreta |
| BRbv276-09 | MK910419 | bovine | 2009 | Rondonopolis |
| BRbv319-09 | MK910524 | bovine | 2009 | LambariD'Oeste |
| BRbv338-09 | MK910403 | bovine | 2009 | AltoParaguai |
| BRbv350-09 | MK910492 | bovine | 2009 | NovaMarilandia |
| BRbv360-09 | MK910412 | bovine | 2009 | GloriaD'Oeste |
| BRbv390-09 | MK910506 | bovine | 2009 | NovoSaoJoaquim |
| BRbv410-09 | MK910521 | bovine | 2009 | Rondonopolis |
| BRbv442-09 | MK910411 | bovine | 2009 | NovaStaHelena |
| BRbv444-09 | MK910531 | bovine | 2009 | GeneralCarneiro |
| BRbv445-09 | MK910482 | bovine | 2009 | Indiavai |
| BRbv447-09 | MK910481 | bovine | 2009 | FigueiropolisD'Oeste |
| BRbv448-09 | MK910489 | bovine | 2009 | SantoAfonso |
| BRbv449-09 | MK910480 | bovine | 2009 | FigueiropolisD'Oeste |
| BRbv466-09 | MK910520 | bovine | 2009 | StoAntoninoLeverger |
| BRbv01-10 | MK910529 | bovine | 2010 | NovoSaoJoaquim |
| BRbv03-10 | MK910474 | bovine | 2010 | SaoJosequineuatroMarcos |
| BRbv09-10 | MK910447 | bovine | 2010 | Arenapolis |
| BRbv10-10 | MK910429 | bovine | 2010 | Jauru |
| BRbv17-10 | MK910518 | bovine | 2010 | PedraPreta |
| BRbv19-10 | MK910523 | bovine | 2010 | PedraPreta |
| BRbv47-10 | MK910435 | bovine | 2010 | Diamantino |
| BRbv77-10 | MK910448 | bovine | 2010 | AltoParaguai |
| BRbv80-10 | MK910525 | bovine | 2010 | Rondonopolis |
| BRbv87-10 | MK910503 | bovine | 2010 | Poxoreu |
| BRbv96-10 | MK910486 | bovine | 2010 | PortoEsperidiao |
| BRbv105-10 | MK910428 | bovine | 2010 | Jauru |
| BRbv109-10 | MK910528 | bovine | 2010 | NovoSaoJoaquim |
| BRbv134-10 | MK910512 | bovine | 2010 | GeneralCarneiro |
| BRbv140-10 | MK910431 | bovine | 2010 | PortoEsperidiao |
| BRbv185-10 | MK910427 | bovine | 2010 | Araputanga |
| BRbv196-10 | MK910485 | bovine | 2010 | PontesELacerda |
| BRbv211-10 | MK910426 | bovine | 2010 | ValeDoSaoDomingos |
| BRbv227-10 | MK910440 | bovine | 2010 | Cuiaba |
| BRbv251-10 | MK910484 | bovine | 2010 | GloriaD'Oeste |
| BRbv318-10 | MK910425 | bovine | 2010 | PortoEsperidiao |
| BRbv319-10 | MK910410 | bovine | 2010 | PortoEsperidiao |
| BRbv329-10 | MK910409 | bovine | 2010 | Jauru |
| BRbv331-10 | MK910418 | bovine | 2010 | MirassolD'Oeste |
| BRbv346-10 | MK910408 | bovine | 2010 | MirassolD'Oeste |
| BRbv353-10 | MK910424 | bovine | 2010 | PortoEsperidiao |
| BRbv379-10 | MK910538 | bovine | 2010 | Diamantino |
| BRbv387-10 | MK910454 | bovine | 2010 | PontesELacerda |
| BRbv389-10 | MK910441 | bovine | 2010 | Cuiaba |
| BRbv419-10 | MK910399 | bovine | 2010 | PortoEsperidiao |
| BRbv451-10 | MK910509 | bovine | 2010 | AltoTaquari |
| BRbv470-10 | MK910510 | bovine | 2010 | AltoTaquari |
| BRbv482-10 | MK910438 | bovine | 2010 | Cuiaba |
| BRbv18-11 | MK910493 | bovine | 2011 | Caceres |
| BRbv62-11 | MK910511 | bovine | 2011 | PrimaveraDoLeste |
| BRbv86-11 | MK910530 | bovine | 2011 | GeneralCarneiro |
| BRbv108-11 | MK910422 | bovine | 2011 | PontesELacerda |
| BRbv131-11 | MK910470 | bovine | 2011 | Jauru |
| BRbv134-11 | MK910430 | bovine | 2011 | PontesELacerda |
| BRbv141-11 | MK910407 | bovine | 2011 | PontesELacerda |
| BRbv142-11 | MK910416 | bovine | 2011 | PontesELacerda |
| BRbv144-11 | MK910460 | bovine | 2011 | Caceres |
| BRbv154-11 | MK910505 | bovine | 2011 | BarraDoGracas |
| BRbv159-11 | MK910495 | bovine | 2011 | PortoEsperidiao |
| BRbv175-11 | MK910494 | bovine | 2011 | PortoEsperidiao |
| BRbv177-11 | MK910476 | bovine | 2011 | Itauba |
| BRbv182-11 | MK910475 | bovine | 2011 | PontesELacerda |
| BRbv186-11 | MK910463 | bovine | 2011 | PontesELacerda |
| BRbv195-11 | MK910462 | bovine | 2011 | PortoEseridiao |
| BRbv196-11 | MK910434 | bovine | 2011 | Jangada |
| BRbv198-11 | MK910406 | bovine | 2011 | PontesELacerda |
| BRbv200-11 | MK910405 | bovine | 2011 | PontesELacerda |
| BRbv201-11 | MK910473 | bovine | 2011 | PontesELacerda |
| BRbv207-11 | MK910456 | bovine | 2011 | PontesELacerda |
| BRbv221-11 | MK910472 | bovine | 2011 | PortoEsperidiao |
| BRbv222-11 | MK910420 | bovine | 2011 | PontesELacerda |
| BRbv230-11 | MK910415 | bovine | 2011 | PontesELacerda |
| BRbv239-11 | MK910461 | bovine | 2011 | Caceres |
| BRbv245-11 | MK910457 | bovine | 2011 | PontesELacerda |
| BRbv246-11 | MK910458 | bovine | 2011 | PontesELacerda |
| BRbv267-11 | MK910414 | bovine | 2011 | PontesELacerda |
| BRbv268-11 | MK910465 | bovine | 2011 | PortoEsperidiao |
| BRbv281-11 | MK910459 | bovine | 2011 | PontesELacerda |
| BRbv305-11 | MK910496 | bovine | 2011 | Caceres |
| BRbv319-11 | MK910413 | bovine | 2011 | PontesELacerda |
| BRbv320-11 | MK910498 | bovine | 2011 | Juscimeira |
| BRbv331-11 | MK910471 | bovine | 2011 | PontesELacerda |
| BRbv191-10 | MK910542 | bovine | 2010 | Araguaina |
| BRbv267-10 | MK910551 | bovine | 2010 | Araguaia |
| BRbv299-10 | MK910557 | bovine | 2010 | NovoSantoAntonio |
| BRbv316-10 | MK910541 | bovine | 2010 | NovaXavantina |
| BRbv1-11 | MK910550 | bovine | 2011 | BarraDoGracas |
| BRbv19-11 | MK910555 | bovine | 2011 | RibeiraoCasalheira |
| BRbv123-11 | MK910556 | bovine | 2011 | RibeiraoCascalheira |
| BRbv197-11 | MK910549 | bovine | 2011 | Araguaiana |
| BRbv218-11 | MK910553 | bovine | 2011 | RibeiraoCascalheira |
| BRbv220-11 | MK910554 | bovine | 2011 | RibeiraoCascalheira |
| BRbv233-11 | MK910539 | bovine | 2011 | BarraDoGarcas |
| BRbv241-11 | MK910548 | bovine | 2011 | Araguaiana |
| BRbv244-11 | MK910547 | bovine | 2011 | Araguaiana |
| BRbv247-11 | MK910546 | bovine | 2011 | BarraDoGracas |
| BRbv249-11 | MK910545 | bovine | 2011 | BarraDoGracas |
| BRbv290-11 | MK910544 | bovine | 2011 | BarraDoGracas |
| BRbv303-11 | MK910543 | bovine | 2011 | Campinapolis |
| BRbv329-11 | MK910552 | bovine | 2011 | BarraDoGracas |
| BRbv46-08 | MK910561 | bovine | 2008 | NovaMutum |
| BRbv54-08 | MK910564 | bovine | 2008 | NovaUbirata |
| BRbv95-08 | MK910570 | bovine | 2008 | Guaranta |
| BRbv102-08 | MK910565 | bovine | 2008 | NovaUbirata |
| BRbv168-08 | MK910575 | bovine | 2008 | NovaCanaaDoNorte |
| BRbv216-08 | MK910579 | bovine | 2008 | Colider |
| BRbv190-09 | MK910578 | bovine | 2009 | NovaCanaaDoNorte |
| BRbv258-09 | MK910569 | bovine | 2009 | VilaBelaSsTrinidade |
| BRbv21-10 | MK910568 | bovine | 2010 | AguaBoa |
| BRbv351-10 | MK910574 | bovine | 2010 | NovoMundo |
| BRbv37-10 | MK910559 | bovine | 2010 | Araguaiana |
| BRbv109-11 | MK910576 | bovine | 2011 | NovoMundo |
| BRbv125-11 | MK910562 | bovine | 2011 | Itauba |
| BRbv158-11 | MK910566 | bovine | 2011 | Matupa |
| BRbv176-11 | MK910567 | bovine | 2011 | PontesELacerda |
| BRbv235-11 | MK910577 | bovine | 2011 | NovaGuarita |
| BRbv283-11 | MK910580 | bovine | 2011 | Canarana |
| BRbv285-11 | MK910573 | bovine | 2011 | Sinop |
| BRbv286-11 | MK910572 | bovine | 2011 | Sinop |
| BRbv118-11 | MK910563 | bovine | 2011 | Altamira |
| BRkj | deposited* | kinkajou | 2010 | Mato Grosso state |
| BRhr71-08 | MK910527 | equine | 2008 | Rondonopolis |
| BRhr120-08 | MK910560 | equine | 2008 | Lucas Do Rio Verde |
| BRhr219-08 | MK910526 | equine | 2008 | Nova Guarita |
| BRhr561-08 | MK910450 | equine | 2008 | Rondonopolis |
| BRhr150-09 | MK910433 | equine | 2009 | Indiavai |
| BRhr211-09 | MK910442 | equine | 2009 | Nobres |
| BRhr183-10 | MK910508 | equine | 2010 | Alto Taquari |
| BRhr38-11 | MK910537 | equine | 2011 | Diamantino |
| BRhr83-11 | MK910423 | equine | 2011 | Pontes Elacerda |
| BRhr101-11 | MK910558 | equine | 2011 | Ribeirao Cascalheira |
| BRhr124-11 | MK910455 | equine | 2011 | Pontes Elacerda |
| BRhr126-11 | MK910417 | equine | 2011 | Pontes Elacerda |
| BRhr155-11 | MK910452 | equine | 2011 | Santo Afonso |
| BRhr217-11 | MK910421 | equine | 2011 | Pontes Elacerda |
| BRhr229-11 | MK910483 | equine | 2011 | Pontes Elacerda |
| BRhr266-11 | MK910466 | equine | 2011 | Pontes Elacerda |
| BRhr279-11 | MK910540 | equine | 2011 | Barra Do Gracas |
| BRhr280-11 | MK910453 | equine | 2011 | Santo Afonso |
| BRhr317-11 | MK910571 | equine | 2011 | Arenapolis |
| BRhr343-11 | MK910497 | equine | 2011 | Caceres |
