# Supplementary material for: Unexpected rabies variant identified in kinkajou (Potos flavus), Mato Grosso, Brazil
Source: Emerg Microbes Infect. 2020 May 14;9(1):851–4. doi: 10.1080/22221751.2020.1759380 (PMC7269027; doi:10.1080/22221751.2020.1759380)
Supplement: Supplemental Material [file TEMI_A_1759380_SM7515.zip › 1759380/Appendix Table 2.docx]

| **Sample ID** | **GenBank accession number** | **Host** | **Host scientific name** | **Year** | **Location** |
| --- | --- | --- | --- | --- | --- |
| IP-2990-13 | KM594041 | Vampire bat | *Desmodus rotondus* | 2013 | Brazil |
| IP-2992-13 | KM594040 | Vampire bat | *Desmodus rotondus* | 2013 | Brazil |
| IP-2991-13 | KM594042 | Vampire bat | *Desmodus rotondus* | 2013 | Brazil |
| IP-512-09 | KM594026 | Argentine brown bat | *Eptesicus furinalis* | 2009 | Brazil |
| IP-230-10 | KM594027 | Argentine brown bat | *Eptesicus furinalis* | 2010 | Brazil |
| IP-346-10 | KM594028 | Argentine brown bat | *Eptesicus furinalis* | 2010 | Brazil |
| IP-3208-06 | KM594029 | Argentine brown bat | *Eptesicus furinalis* | 2006 | Brazil |
| IP-1400-10 | KM594030 | Black myotis | *Myotis nigricans* | 2010 | Brazil |
| IP-163-10 | KM594031 | Black myotis | *Myotis nigricans* | 2010 | Brazil |
| IP-497-10 | KM594032 | Black myotis | *Myotis nigricans* | 2010 | Brazil |
| IP-6634-08 | KM594023 | Common marmoset | *Callithrix jacchus* | 2008 | Brazil |
| IP-1770-12 | KM594024 | Common marmoset | *Callithrix jacchus* | 2012 | Brazil |
| IP-5402-07 | KM594025 | Common marmoset | *Callithrix jacchus* | 2007 | Brazil |
| 86117BRE | KX148109 | Dog | *Canis lupus familiaris* | 1986 | Brazil |
| P17 | AB618035 | Cow | *Bos taurus* | 2006 | Brazil |
| P18 | AB618036 | Domestic sheep | *Ovis aries* | 2007 | Brazil |
| BRmk1358 | AB810256 | Tufted capuchin monkey | *Cebus apella* | 2010 | Brazil |
| IP-4005-12 | KM594043 | Cow | *Bos taurus* | 2012 | Brazil |
| IP-3176-09 | KM594037 | Mexican free-tailed bat | *Tadarida brasiliensis* | 2009 | Brazil |
| IP-1586-10 | KM594038 | Mexican free-tailed bat | *Tadarida brasiliensis* | 2010 | Brazil |
| IP-4431-10 | KM594033 | Mexican free-tailed bat | *Tadarida brasiliensis* | 2011 | Brazil |
| IP-350-10 | KM594034 | Broad-eared bat | *Nyctinomops laticaudatus* | 2010 | Brazil |
| IP-542-10 | KM594036 | Broad-eared bat | *Nyctinomops laticaudatus* | 2010 | Brazil |
| IP-412-10 | KM594035 | Broad-eared bat | *Nyctinomops laticaudatus* | 2010 | Brazil |
| MP19 | AB618032 | Velvety free-tailed bat | *Molossus molossus* | 2006 | Brazil |
| MPVI | AB618034 | Velvety free-tailed bat | *Molossus molossus* | 2007 | Brazil |
| BR-BAT34 | AB608731 | Southern yellow bat | *Lasiurus ega* | na | Brazil |
| 97004ARG | KX148269 | Mexican free-tailed bat | *Tadarida brasiliensis* | 1997 | Argentina |
| MEXSK3644 | JQ685929 | Eastern spotted skunk | *Spilogale potorius* | 2009 | Mexico |
| MEXSK13938 | JQ685954 | Eastern spotted skunk | *Spilogale potorius* | 2007 | Mexico |
| Coati-3639 | JQ685963 | White-nosed coati | *Nasua narica* | 2009 | Mexico |
| 3634DR | JQ685936 | Bovine | *Bos taurus* | 2009 | Mexico |
| 3645DR | JQ685953 | Human | *Homo sapiens* | 2009 | Mexico |
| 09035FRA | KX148100 | Stenodermatine bat | *Stenodermatinae spp* | 2009 | French Guiana |
| AT6 | KU523255 | Vampire bat | *Desmodus rotondus* | 2010 | French Guiana |
| 90001FRA | KX148268 | Dog | *Canis lupus familiaris* | 1990 | French Guiana |
| PERMDIKN1766-07 | JX648546 | Kinkajou | *Potos flavus* | 2007 | Peru |
| ON-2000-1818 | KY026434 | Raccoon | *Procyon lotor* | 2000 | Canada |
| ON-2004-7702 | KY026476 | Raccoon | *Procyon lotor* | 2004 | Canada |
| NY-2011-1548 | KY026421 | Raccoon | *Procyon lotor* | 2011 | USA |
| VT-2008-0237 | KY026482 | Raccoon | *Procyon lotor* | 2008 | USA |
| VT-2006-0259 | KY026479 | Striped skunk | *Mephitis mephitis* | 2006 | USA |
| A10-0514 | JQ685938 | Striped skunk | *Mephitis mephitis* | 2009 | USA |
| A10-0512 | JQ685968 | Striped skunk | *Mephitis mephitis* | 2009 | USA |
| FL385 | JQ685905 | Mexican free-tailed bat | *Tadarida brasiliensis* | 2003 | USA |
| 2396 | JQ685937 | Ringtail | *Bassariscus astutus* | 2009 | USA |
| 2402 | JQ685896 | Gray fox | *Urocyon cinereoargenteus* | 2009 | USA |
| A093500 | JQ685898 | Big brown bat | *Eptesicus fuscus* | 2009 | USA |
| SM3844 | JQ685974 | Big brown bat | *Eptesicus fuscus* | 1995 | USA |
| AZBAT-6763 | JQ685913 | Big brown bat | *Eptesicus fuscus* | 1985 | USA |
| CA100 | JQ685909 | Big brown bat | *Eptesicus fuscus* | 2005 | USA |
| SM4871 | JQ685923 | Big brown bat | *Eptesicus fuscus* | 1999 | USA |
| AZ2408 | JQ685926 | Big brown bat | *Eptesicus fuscus* | 2005 | USA |
| AZBAT-7453 | JQ685956 | Big brown bat | *Eptesicus fuscus* | 1975 | USA |
| SM5442 | JQ685897 | Big brown bat | *Eptesicus fuscus* | 2001 | USA |
| AZ10-140 | JQ685961 | Big brown bat | *Eptesicus fuscus* | 2010 | USA |
| SM4872 | JQ685960 | Big brown bat | *Eptesicus fuscus* | 2001 | USA |
| SM4862 | JQ685946 | Big brown bat | *Eptesicus fuscus* | 1999 | USA |
| AZ10-144 | JQ685951 | Big brown bat | *Eptesicus fuscus* | 2010 | USA |
| CA04148 | JQ685903 | Big brown bat | *Eptesicus fuscus* | 2004 | USA |
| WA0173 | JQ685931 | Big brown bat | *Eptesicus fuscus* | 2000 | USA |
| AZBAT-65094 | JQ685942 | Big brown bat | *Eptesicus fuscus* | 1981 | USA |
| EF | JQ685920 | Big brown bat | *Eptesicus fuscus* | 1984 | USA |
| WAEF03 | JQ685925 | Big brown bat | *Eptesicus fuscus* | 2004 | USA |
| AZ4490 | JQ685955 | Yuma myotis | *Myotis yumanensis* | 2005 | USA |
| A02-2971 | JQ685952 | Western pipistrelle | *Parastrellus hesperus* | 2002 | USA |
| A02-2972 | JQ685965 | Western pipistrelle | *Parastrellus hesperus* | 2002 | USA |
| AZ3003 | JQ685971 | Pallid bat | *Antrozous pallidus* | 2009 | USA |
| TN310 | JQ685947 | Hoary bat | *Lasiurus cinereus* | 2004 | USA |
| NJ2262 | JQ685919 | Eastern red bat | *Lasiurus borealis* | 2005 | USA |
| FL769 | JQ685900 | Seminole bat | *Lasiurus seminolus* | 2003 | USA |
| TX5960 | JQ685910 | Western yellow bat | *Lasiurus xanthinus* | 2002 | USA |
| TN209 | JQ685902 | Eastern red bat | *Lasiurus borealis* | 2005 | USA |
| WA1185 | JQ685895 | Silver-haired bat | *Lasionycteris noctivagans* | 2003 | USA |
| TN186 | JQ685922 | Tricolored bat | *Perimyotis subflavus* | 2005 | USA |
| FL1078 | JQ685921 | Southeastern myotis | *Myotis austroriparius* | 2001 | USA |
| TX4904 | JQ685915 | Northern yellow bat | *Lasiurus intermedius* | 2002 | USA |
| FL1010 | JQ685916 | Northern yellow bat | *Lasiurus intermedius* | 2002 | USA |
| SM5470 | JQ685966 | Striped skunk | *Mephitis mephitis* | 2001 | USA |
| SM1545 | JQ685941 | Striped skunk | *Mephitis mephitis* | 2005 | USA |
| A11-1043 | JQ685973 | Coyote | *Canis latrans* | 2011 | USA |
| CO-Coyot-2010 | JQ685917 | Coyote | *Canis latrans* | 2010 | USA |
| OR05455 | JQ685948 | Gray fox | *Urocyon cinereoargenteus* | 2010 | USA |
| OR58 | JQ685977 | Gray fox | *Urocyon cinereoargenteus* | 2010 | USA |
| OR05506 | JQ685918 | Gray fox | *Urocyon cinereoargenteus* | 2010 | USA |
| SM5950 | JQ685933 | Gray fox | *Urocyon cinereoargenteus* | 2004 | USA |
| SM6709 | JQ685945 | Cat | *Felis catus* | 2005 | USA |
| RV9 EBLV-1 | EF157976 | Serotine bat | *Eptesicus serotinus* | 1968 | Germany |
| RV13333 EBLV-2 | EF157977 | Human | *Homo sapiens* | 2002 | United Kingdom |
| RV3267 GBLV | KU244267 | Indian flying fox | *Pteropus giganteus* | 2015 | Sri Lanka |
