# Supplementary material for: Unexpected rabies variant identified in kinkajou (Potos flavus), Mato Grosso, Brazil
Source: Emerg Microbes Infect. 2020 May 14;9(1):851–4. doi: 10.1080/22221751.2020.1759380 (PMC7269027; doi:10.1080/22221751.2020.1759380)
Supplement: Supplemental Material [file TEMI_A_1759380_SM7515.zip › 1759380/Appendix Table 3.docx]

| **Sample ID** | **GenBank accession number** | **Host** | **Host scientific name** | **Year** | **Location** |
| --- | --- | --- | --- | --- | --- |
| BRmk1358 | AB810256 | Tufted capuchin monkey | *Cebus apella* | 2010 | Brazil |
| 392_10 | JQ071970 | Cattle | *Bos taurus* | 2010 | Brazil |
| A845_2007_CORR | KF864380 | Maned wolf | *Chrysocyon brachyurus* | 2007 | Argentina |
| V587 | AY854587 | Vampire bat | *Desmodus rotondus* | 1996 | Mexico |
| V917 | AY877433 | Vampire bat | *Desmodus rotondus* | 1990 | Mexico |
| V920 | AY877435 | Vampire bat | *Desmodus rotondus* | 1993 | Mexico |
| IP9851Turiacu_Maranhao/2005 | EF363739 | Human | *Homo sapiens* | 2005 | Brazil |
| IP3072Portel_Para/2004 | EF363747 | Human | *Homo sapiens* | 2004 | Brazil |
| NMBATDic2007insteadofTB1 | FJ228486 | Mexican free-tailed bat | *Tadarida brasiliensis* | 2007 | USA |
| NewMexicoDG2006 | FJ228487 | Dog | *Canis lupus familiaris* | 2006 | USA |
| 3878Mxslpbv04 | FJ228488 | Cow | *Bos taurus* | 2004 | Mexico |
| 3858Mxhgoov03 | FJ228489 | Sheep | *Ovis aries* | 2003 | Mexico |
| 3888Mxbvtamp03 | FJ228491 | Cow | *Bos taurus* | 2003 | Mexico |
| 2153Salvhm02 | FJ228492 | Human | *Homo sapiens* | 2002 | El Salvador |
| 3901Mxmichbv03 | FJ228493 | Cow | *Bos taurus* | 2003 | Mexico |
| 00PINHALZINHO3141B | FJ649134 | Cattle | *Bos taurus* | 2000 | Brazil |
| 0423_06 | GQ915419 | Cattle | *Bos taurus* | 2006 | Brazil |
| 4488Mxbvtamp02 | GU991824 | Bovine | *Bos taurus* | 2002 | Mexico |
| 265QuintanaRoobv9265QuintanaRoobv99 | GU991826 | Bovine | *Bos taurus* | 1999 | Mexico |
| 1475HmEdoMex99 | GU991827 | Human | *Homo sapiens* | 1999 | Mexico |
| 3902bovineMichoacan2003 | GU991828 | Bovine | *Bos taurus* | 2003 | Mexico |
| 3256MxChihbat99 | GU991830 | Mexican free-tailed bat | *Tadarida brasiliensis* | 1999 | Mexico |
| A09_105AL2009 | GU991832 | Mexican free-tailed bat | *Tadarida brasiliensis* | 2009 | USA |
| BR-DR1 | AB201803 | Vampire bat | *Desmodus rotondus* | 2000 | Brazil |
| BR-DR8 | AB297635 | Vampire bat | *Desmodus rotondus* | 2003 | Brazil |
| 98JOANOPOLIS4307B | FJ649069 | Cattle | *Bos taurus* | 1998 | Brazil |
| 99EXTREMA2417B | FJ649082 | Cattle | *Bos taurus* | 1999 | Brazil |
| 00ATIBAIA81B | FJ649105 | Cattle | *Bos taurus* | 2000 | Brazil |
| 01MORUNGABA272B | FJ649160 | Cattle | *Bos taurus* | 2001 | Brazil |
| 07AndradasMG1139B | GQ160910 | Cattle | *Bos taurus* | 2007 | Brazil |
| 07SocorroSP4548B | GQ160919 | Cattle | *Bos taurus* | 2007 | Brazil |
| 08BeloHorizonteMG162B | GQ160921 | Cattle | *Bos taurus* | 2008 | Brazil |
| 08JoanopolisSP3357E | GQ160935 | Equine | *Equus ferus caballus* | 2008 | Brazil |
| 08TresCoracoesMG1049B | GQ160956 | Cattle | *Bos taurus* | 2008 | Brazil |
| 08VargemSP1696E | GQ160957 | Equine | *Equus ferus caballus* | 2008 | Brazil |
| 0154_03 | GQ915406 | Cattle | *Bos taurus* | 2003 | Brazil |
| 229_04 | GQ915408 | Cattle | *Bos taurus* | 2004 | Brazil |
| brdrusp100/07 | GU592648 | Vampire bat | *Desmodus rotondus* | 2007 | Brazil |
| 0154_03 | HM585151 | Cattle | *Bos taurus* | 2003 | Brazil |
| 229_04 | HM585153 | Cattle | *Bos taurus* | 2004 | Brazil |
| 262_04 | HM585158 | Cattle | *Bos taurus* | 2004 | Brazil |
| 05070_02 | HM585177 | Cattle | *Bos taurus* | 2002 | Brazil |
| 451_05_Bov_SP | JF682414 | Cattle | *Bos taurus* | 2005 | Brazil |
| 3924_Bov_SP | JF682415 | Cattle | *Bos taurus* | 2005 | Brazil |
| 3833_04_Bov_SP | JF682421 | Cattle | *Bos taurus* | 2004 | Brazil |
| 11817_04_Bov_SP | JF682425 | Cattle | *Bos taurus* | 2004 | Brazil |
| 9001GUY | U22478 | Dog | *Canis lupus familiaris* | 1990 | French Guiana |
| 9001FRA | EU293113 | Dog | *Canis lupus familiaris* | 1990 | French Guiana |
| 9905FRA | KT276367 | Cattle | *Bos taurus* | 1999 | French Guiana |
| 9703FRA | KT276365 | Cattle | *Bos taurus* | 1999 | French Guiana |
| RV1814 | KF424538 | Cattle | *Bos taurus* | unknown | Guiana |
| PERCUSBV38910-03 | JX648444 | Cattle | *Bos taurus* | 2003 | Peru |
| PERMDIHM1710-07 | JX648434 | Human | *Homo sapiens* | 2007 | Peru |
| PERCUSHM8714-07 | JX648443 | Human | *Homo sapiens* | 2007 | Peru |
| PERCUSBV38910-03 | JX648444 | Cow | *Bos taurus* | 2003 | Peru |
| A709_2006_MIS | KF864329 | Cow | *Bos taurus* | 2006 | Argentina |
| A704_2006_MIS | KF864327 | Cow | *Bos taurus* | 2006 | Argentina |
| IP6767U/08 | EU981917 | Cow | *Bos taurus* | 2008 | Uruguay |
| IP6785U/07 | EU981931 | Vampire bat | *Desmodus rotondus* | 2007 | Uruguay |
| BR-AL4 | AB201802 | Great fruit-eating bat | *Artibeus lituratus* | 2002 | Brazil |
| IP3066Portel_Para/2004 | EF363742 | Human | *Homo sapiens* | 2004 | Brazil |
| IP3522Portel_Para/2004 | EF363748 | Human | *Homo sapiens* | 2004 | Brazil |
| 08MariliaSP1700B | GQ160938 | Cattle | *Bos taurus* | 2008 | Brazil |
| 3250_05_Art_SP | JF682392 | Neotropical fruit bat | *Artibeus sp.* | 2005 | Brazil |
| 7270_05_Art_SP | JF682406 | Great fruit-eating bat | *Artibeus lituratus* | 2005 | Brazil |
| DR.Td2 | AF351852 | Cattle | *Bos taurus* | 1995 | Trinidad |
| IP2293Pastaza_Ecuador/2005 | EF363727 | Human | *Homo sapiens* | 2005 | Ecuador |
| IP2294Pastaza_Ecuador/2005 | EF363728 | Human | *Homo sapiens* | 2005 | Ecuador |
| IP4405EQ/07BOV | HM368176 | Cattle | *Bos taurus* | 2007 | Ecuador |
| IP4407EQ/07BOV | HM368178 | Cattle | *Bos taurus* | 2007 | Ecuador |
| IP4403EQ/07BOV | HM368179 | Cattle | *Bos taurus* | 2007 | Ecuador |
| BV1/06 | JF693466 | Cattle | *Bos taurus* | 2006 | Colombia |
| BV7/97 | JF693467 | Cattle | *Bos taurus* | 1997 | Colombia |
| BV1/03 | JF693468 | Cattle | *Bos taurus* | 2003 | Colombia |
| H02/07 | JF693470 | Human | *Homo sapiens* | 2007 | Colombia |
| PERAMAPG2034-05 | JX648408 | Pig | *Sus scrofa* | 2005 | Peru |
| PERSMABV33842-02 | JX648413 | Cattle | *Bos taurus* | 2002 | Peru |
| PERHUABV39200-03 | JX648414 | Cattle | *Bos taurus* | 2003 | Peru |
| PERHUABV41340-03 | JX648415 | Cattle | *Bos taurus* | 2003 | Peru |
| PERPASBV13969-07 | JX648421 | Cattle | *Bos taurus* | 2007 | Peru |
| PERAYADK50031-05 | JX648422 | Donkey |  | 2005 | Peru |
| PERSMABV8429-07 | JX648423 | Cattle | *Bos taurus* | 2007 | Peru |
| PERSMAHR35351-02 | JX648424 | Horse | *Equus ferus caballus* | 2002 | Peru |
| PERSMAHR17048-04 | JX648429 | Horse | *Equus ferus caballus* | 2004 | Peru |
| PERAMABV51713-06 | JX648430 | Cattle | *Bos taurus* | 2006 | Peru |
| BRhr31 | AB083804 | Horse | *Equus ferus caballus* | 1998 | Brazil |
| BRbv56 | AB675603 | Cattle | *Bos taurus* | 1998 | Brazil |
| BRbv403 | AB675621 | Cattle | *Bos taurus* | 2002 | Brazil |
| BRvmbt46 | AB083815 | Vampire bat | *Desmodus rotondus* | unknown | Brazil |
| BRbv50 | AB083818 | Cattle | *Bos taurus* | 1990 | Brazil |
| BR-AL1 | AB117969 | Great fruit-eating bat | *Artibeus lituratus* | 1998 | Brazil |
| BR-A2 | AB297628 | Neotropical fruit bat | *Artibeus sp.* | 2004 | Brazil |
| BR-DR9 | AB297636 | Vampire bat | *Desmodus rotondus* | 2000 | Brazil |
| 97MORUNGABA248B | FJ649044 | Cattle | *Bos taurus* | 1997 | Brazil |
| 98CAJURU5331B | FJ649075 | Cattle | *Bos taurus* | 1998 | Brazil |
| 99SOCORRO1989B | FJ649079 | Cattle | *Bos taurus* | 1999 | Brazil |
| 07MococaSP2647E | GQ160914 | Horse | *Equus ferus caballus* | 2007 | Brazil |
| 08SalesopolisSP10166B | GQ160943 | Cattle | *Bos taurus* | 2008 | Brazil |
| 065_03 | HM585149 | Cattle | *Bos taurus* | 2003 | Brazil |
| 243_04 | HM585154 | Cattle | *Bos taurus* | 2004 | Brazil |
| 0424_06 | HM585165 | Cattle | *Bos taurus* | 2006 | Brazil |
| 0469_01 | HM585170 | Cattle | *Bos taurus* | 2001 | Brazil |
| 0697_02 | HM585172 | Sheep | *Ovis aries* | 2002 | Brazil |
| 3738_05_Art_SP | JF682395 | Neotropical fruit bat | *Artibeus sp.* | 2005 | Brazil |
| 6734_05_Art_SP | JF682399 | Great fruit-eating bat | *Artibeus lituratus* | 2005 | Brazil |
| 6956_05_Art_SP | JF682400 | Neotropical fruit bat | *Artibeus sp.* | 2005 | Brazil |
| PEHM3230 | AF045166 | Human | *Homo sapiens* | 1996 | Peru |
| A592_2005_MIS | KF864296 | Bovine | *Bos taurus* | 2005 | Argentina |
| BRvmbt41 | AB083812 | Vampire bat | *Desmodus rotondus* | unknown | Brazil |
| natural-host Desmodus rotundus | AF070449 | Vampire bat | *Desmodus rotondus* | unknown | Brazil |
| BRvmbt33 | AB083806 | Vampire bat | *Desmodus rotondus* | unknown | Brazil |
| BRvmbt34 | AB083807 | Vampire bat | *Desmodus rotondus* | 1998 | Brazil |
| BRsp35 | AB083808 | Sheep | *Ovis aries* | 1992 | Brazil |
| BR-AL6 | AB297630 | Great fruit-eating bat | *Artibeus lituratus* | 2001 | Brazil |
| BR-DR7 | AB297634 | Vampire bat | *Desmodus rotondus* | 1997 | Brazil |
| 399 | EF428576 | Vampire bat | *Desmodus rotondus* | 2006 | Brazil |
| 97SALESOPOLIS719B | FJ649047 | Cattle | *Bos taurus* | 1997 | Brazil |
| 97SALESOPOLIS1286B | FJ649050 | Cattle | *Bos taurus* | 1997 | Brazil |
| 98SJBOAVISTA4992B | FJ649072 | Cattle | *Bos taurus* | 1998 | Brazil |
| 0319_02 | GQ915415 | Horse | *Equus ferus caballus* | 2002 | Brazil |
| 0337_06 | GQ915416 | Horse | *Equus ferus caballus* | 2006 | Brazil |
| 004_04 | GQ915433 | Horse | *Equus ferus caballus* | 2004 | Brazil |
| 0319_02 | HM585160 | Horse | *Equus ferus caballus* | 2002 | Brazil |
| 0337_06 | HM585161 | Horse | *Equus ferus caballus* | 2006 | Brazil |
| BRbv36 | AB083809 | Cattle | *Bos taurus* | 1998 | Brazil |
| BRbv38 | AB083810 | Cattle | *Bos taurus* | 1999 | Brazil |
| BRbv39 | AB083811 | Cattle | *Bos taurus* | 1999 | Brazil |
| BR-Pbv12 | AB206431 | Cattle | *Bos taurus* | unknown | Brazil |
| BRhr1196 | AB623076 | Horse | *Equus ferus caballus* | 2007 | Brazil |
| DR.Braz | AF351847 | Cattle | *Bos taurus* | 1986 | Brazil |
| brbvusp01/06 | GU592649 | Cattle | *Bos taurus* | 2006 | Brazil |
| 86118BRE | U22479 | Vampire bat | *Desmodus rotondus* | 1985 | Brazil |
| BR-DR10 | AB297637 | Vampire bat | *Desmodus rotondus* | 2005 | Brazil |
| BR-DR11 | AB297638 | Vampire bat | *Desmodus rotondus* | 2005 | Brazil |
| BR-DR12 | AB297639 | Vampire bat | *Desmodus rotondus* | 2005 | Brazil |
| BR-DR14 | AB297641 | Vampire bat | *Desmodus rotondus* | 2005 | Brazil |
| BR-DR16 | AB297642 | Vampire bat | *Desmodus rotondus* | 2005 | Brazil |
| BR-DR20 | AB297645 | Vampire bat | *Desmodus rotondus* | 2006 | Brazil |
| P18 | AB618036 | Sheep | *Ovis aries* | 2007 | Brazil |
| BRbv804 | AB675628 | Cattle | *Bos taurus* | 2005 | Brazil |
| BRbv827 | AB675629 | Cattle | *Bos taurus* | 2005 | Brazil |
| 08PedregulhoSP180B | GQ160940 | Cattle | *Bos taurus* | 2008 | Brazil |
| H01/08 | JF693456 | Human | *Homo sapiens* | 2008 | Colombia |
| H02/08 | JF693457 | Human | *Homo sapiens* | 2008 | Colombia |
| CT1/06 | JF693458 | Cat | *Felis catus* | 2006 | Colombia |
| H01/04 | JF693461 | Human | *Homo sapiens* | 2004 | Colombia |
| C07/96 | JF693462 | Dog | *Canis lupus familiaris* | 1996 | Colombia |
| CT1/94 | JF693471 | Cat | *Felis catus* | 1994 | Colombia |
| CT3/96 | JF693472 | Cat | *Felis catus* | 1996 | Colombia |
| H04/95 | JF693475 | Human | *Homo sapiens* | 1995 | Colombia |
| H02/94 | JF693476 | Human | *Homo sapiens* | 1994 | Colombia |
| H03/08 | JF693478 | Human | *Homo sapiens* | 2008 | Colombia |
| FL385 | JQ685905 | Mexican free-tailed bat | *Tadarida brasiliensis* | 2003 | USA |
| 3634DR | JQ685936 | Bovine | *Bos taurus* | 2009 | Mexico |
| 3645DR | JQ685953 | Human | *Homo sapiens* | 2009 | Mexico |
| PERAYABV12690-07 | JX648445 | Bovine | *Bos taurus* | 2007 | Peru |
| PERAYABV13485-07 | JX648446 | Bovine | *Bos taurus* | 2007 | Peru |
| PERAPUBV43925-04 | JX648447 | Bovine | *Bos taurus* | 2004 | Peru |
| PERAYABV14916-05 | JX648448 | Bovine | *Bos taurus* | 2005 | Peru |
| PERAYABV29517-02 | JX648468 | Bovine | *Bos taurus* | 2002 | Peru |
| PERAPUBV249-03 | JX648508 | Bovine | *Bos taurus* | 2003 | Peru |
| PERAPUBV13695-07 | JX648509 | Bovine | *Bos taurus* | 2007 | Peru |
| PERAPUBT10342-06 | JX648524 | Vampire bat | *Desmodus rotondus* | 2006 | Peru |
| PERAYADN47065-05 | JX648542 | Donkey |  | 2005 | Peru |
| PERPASBV14803-06 | JX648543 | Bovine | *Bos taurus* | 2006 | Peru |
| 425_1994_FOR | KF864234 | Vampire bat | *Desmodus rotondus* | 1994 | Argentina |
| IP 2990/13 | KM594041 | Vampire bat | *Desmodus rotondus* | 2013 | Brazil |
| A00_1999_CHA | KF864238 | Bovine | *Bos taurus* | 1999 | Argentina |
| A182_2002_TUC | KF864259 | Bovine | *Bos taurus* | 2002 | Argentina |
| A355_2003_TUC | KF864277 | Horse | *Equus ferus caballus* | 2003 | Argentina |
| A467_2004_SAL | KF864285 | Bovine | *Bos taurus* | 2004 | Argentina |
| A593_2005_SAL | KF864297 | Bovine | *Bos taurus* | 2005 | Argentina |
| A642_2006_FOR | KF864306 | Bovine | *Bos taurus* | 2006 | Argentina |
| A717_2006_SAL | KF864334 | Bovine | *Bos taurus* | 2006 | Argentina |
| A721_2006_FOR | KF864337 | Bovine | *Bos taurus* | 2006 | Argentina |
| A824_2007_SAL | KF864375 | Bovine | *Bos taurus* | 2007 | Argentina |
| A896_2008_CAT | KF864398 | Bovine | *Bos taurus* | 2008 | Argentina |
| A903_2008_CAT | KF864400 | Bovine | *Bos taurus* | 2008 | Argentina |
| A926_2009_CHA | KF864403 | Bovine | *Bos taurus* | 2009 | Argentina |
| A952_2009_SAL | KF864410 | Bovine | *Bos taurus* | 2009 | Argentina |
| A964_2009_CAT | KF864413 | Bovine | *Bos taurus* | 2009 | Argentina |
| IP9854Turiacu_Maranhao/2005 | EF363730 | Human | Homo sapiens | 2005 | Brazil |
| IP5858Turiacu_Maranhao/2005 | EF363732 | Human | Homo sapiens | 2005 | Brazil |
| IP4795Augusto Correa_Para/2005 | EF363749 | Human | Homo sapiens | 2005 | Brazil |
| IP5213Viseu_Para/2004 | EF363750 | Human | Homo sapiens | 2004 | Brazil |
| 484_1996_MIS | KF864235 | Bovine | *Bos taurus* | 1996 | Argentina |
| A01_1999_CORR | KF864239 | Buffalo | *Bubalus bubalis* | 1999 | Argentina |
| A02_1999_CORR | KF864240 | Buffalo | *Bubalus bubalis* | 1999 | Argentina |
| A04_1999_FOR | KF864241 | Bovine | *Bos taurus* | 1999 | Argentina |
| A06_1999_CORR | KF864242 | Bovine | *Bos taurus* | 1999 | Argentina |
| A07_1999_FOR | KF864243 | Bovine | *Bos taurus* | 1997 | Argentina |
| A106_2001_CHA | KF864246 | Bovine | *Bos taurus* | 2001 | Argentina |
| A109_2001_CORR | KF864247 | Bovine | *Bos taurus* | 2001 | Argentina |
| A113_2001_FOR | KF864249 | Bovine | *Bos taurus* | 2001 | Argentina |
| A127_2001_FOR | KF864250 | Bovine | *Bos taurus* | 2001 | Argentina |
| A128_2001_FOR | KF864251 | Bovine | *Bos taurus* | 2001 | Argentina |
| A131_2001_FOR | KF864253 | Bovine | *Bos taurus* | 2001 | Argentina |
| A132_2001_FOR | KF864254 | Bovine | *Bos taurus* | 2001 | Argentina |
| A133_2002_FOR | KF864255 | Bovine | *Bos taurus* | 2002 | Argentina |
| A26_2000_CORR | KF864272 | Bovine | *Bos taurus* | 2000 | Argentina |
| A263_2003_FOR | KF864269 | Bovine | *Bos taurus* | 2003 | Argentina |
| A32_2000_CORR | KF864276 | Bovine | *Bos taurus* | 2000 | Argentina |
| A450_2004_FOR | KF864282 | Bovine | *Bos taurus* | 2004 | Argentina |
| A530_2005_FOR | KF864286 | Bovine | *Bos taurus* | 2005 | Argentina |
| A546_2005_FOR | KF864287 | Bovine | *Bos taurus* | 2005 | Argentina |
| A551_2006_CORR | KF864288 | Bovine | *Bos taurus* | 2006 | Argentina |
| A560_2005_CORR | KF864290 | Bovine | *Bos taurus* | 2005 | Argentina |
| A64_2000_CORR | KF864307 | Bovine | *Bos taurus* | 2000 | Argentina |
| A655_2006_CORR | KF864308 | Bovine | *Bos taurus* | 2006 | Argentina |
| A657_2006_CORR | KF864310 | Bovine | *Bos taurus* | 2006 | Argentina |
| A686_2006_CHA | KF864316 | Bovine | *Bos taurus* | 2006 | Argentina |
| A687_2006_CHA | KF864317 | Bovine | *Bos taurus* | 2006 | Argentina |
| A720_2006_FOR | KF864336 | Bovine | *Bos taurus* | 2006 | Argentina |
| A731_2006_FOR | KF864346 | Bovine | *Bos taurus* | 2006 | Argentina |
| A776_2007_SAL | KF864355 | Bovine | *Bos taurus* | 2007 | Argentina |
| A813_2007_CHA | KF864373 | Bovine | *Bos taurus* | 2007 | Argentina |
| A82_2001_CORR | KF864377 | Human | *Homo sapiens* | 2001 | Argentina |
| A839_2007_FOR | KF864379 | Bovine | *Bos taurus* | 2007 | Argentina |
| A851_2008_FOR | KF864381 | Horse | *Equus ferus caballus* | 2008 | Argentina |
| A858_2008_FOR | KF864382 | Horse | *Equus ferus caballus* | 2008 | Argentina |
| A868_2008_FOR | KF864387 | Bovine | *Bos taurus* | 2008 | Argentina |
| A88_2001_CHA | KF864395 | Bovine | *Bos taurus* | 2001 | Argentina |
| A883_2008_CHA | KF864394 | Bovine | *Bos taurus* | 2008 | Argentina |
| A89_2001_CHA | KF864399 | Bovine | *Bos taurus* | 2001 | Argentina |
| A908_2008_FOR | KF864401 | Bovine | *Bos taurus* | 2008 | Argentina |
| A923_2009_CHA | KF864402 | Bovine | *Bos taurus* | 2009 | Argentina |
| A94_2001_CHA | KF864408 | Bovine | *Bos taurus* | 2001 | Argentina |
| A947_2009_FOR | KF864407 | Bovine | *Bos taurus* | 2009 | Argentina |
| A95_2001_CORR | KF864412 | Bovine | *Bos taurus* | 2001 | Argentina |
| BRbv43 | AB083813 | Bovine | *Bos taurus* | 1999 | Argentina |
| BRbv45 | AB083814 | Bovine | *Bos taurus* | 1999 | Argentina |
| 529_1997_CORR | KF864237 | Bovine | *Bos taurus* | 1997 | Argentina |
| A267_2003_FOR | KF864271 | Bovine | *Bos taurus* | 2003 | Argentina |
| A868_2008_FOR | KF864387 | Bovine | *Bos taurus* | 2008 | Argentina |
| A873_2008_FOR | KF864390 | Bovine | *Bos taurus* | 2008 | Argentina |
