# Supplementary material for: Unexpected rabies variant identified in kinkajou (Potos flavus), Mato Grosso, Brazil
Source: Emerg Microbes Infect. 2020 May 14;9(1):851–4. doi: 10.1080/22221751.2020.1759380 (PMC7269027; doi:10.1080/22221751.2020.1759380)
Supplement: Supplemental Material [file TEMI_A_1759380_SM7515.zip › 1759380/Rocha_et_al_2020_Appendix_after_reviewer_comments_ref_correct_no_track.docx]

**Appendix**

**RNA extraction, RT-PCR and sequencing**

Viral RNA was extracted from brain specimens associated with a rabies epizootic in bovines and equines collected in Brazil in the period 2007-2011, using the Nucleospin RNA II Kit, according to the manufacturer’s instructions (Macherey-Nagel GmbH & Co., Düren, Germany). One hundred microliters of sample suspension were used for the extraction and RNA was eluted in a final volume of 60 μl and stored at -80 °C.

Amplicons of the partial N gene (603bp) were generated performing One-Step RT-PCR as described elsewhere [1] using the Qiagen OneStep RT-PCR kit (Qiagen GmbH, Hilden, Germany), according to the manufacturer’s instructions. They were analyzed by electrophoresis in 1% agarose gel after staining with GelRedTM Nucleic Acid Gel Stain (Biotium, Hayward, CA).

After electrophoresis amplicons were purified with ExoSAP-IT (USB Corporation, Cleveland, OH) and sequenced in both directions using the Big Dye Terminator v3.1 cycle sequencing kit (Applied Biosystems, Foster City, CA, USA). Sequencing clean-up was performed using the Performa DTR Ultra 96-well Kit (Edge BioSystems, Gaithersburg, MD) and samples were subsequently analyzed in a 16-capillary ABI PRISM 3130xl Genetic Analyzer (Applied biosystems, Foster City, CA, USA).

**RNA extraction, library preparation, Illumina MiSeq sequencing**

Total RNA was extracted with the QIAamp Viral RNA mini kit (Qiagen) and then subject to cDNA synthesis with the Maxima H Minus Double-stranded cDNA synthesis kit (ThermoFisher). Library preparation was performed using Nextera XT DNA sample preparation kit and processed on an Illumina MiSeq instrument with MiSeq reagent kit V3 (2x300 bp paired-end [PE] mode; Illumina, San Diego, CA, USA)

**Bioinformatic analysis**

Illumina reads quality was assessed using FastQC v0.11.2 (https://www.bioinformatics.babraham.ac.uk/projects/fastqc/). Raw data were filtered by removing: a) reads with more than 10% of undetermined (“N”) bases; b) reads with more than 100 bases with Q score below 7; c) duplicated paired-end reads. Remaining reads were clipped from Illumina adaptors Nextera XT with scythe v0.991 (https://github.com/vsbuffalo/scythe) and trimmed with sickle v1.33 (https://github.com/najoshi/sickle). Reads shorter than 80 bases or unpaired after previous filters were discarded.

Taxonomic assignment of high-quality reads was carried out using BLAST 2.7.1+ [2] alignment against the integrated NT database (version 12 February 2018) and diamond v0.9.17 [3] alignment against the integrated NR database (version 12 February 2018). Alignment hits with e-values greater than 1×10^-3^ were discarded. Taxonomical level of each read was determined by the lowest common ancestor (LCA)-based algorithm that was implemented in MEGAN ue v6.10.8 [4].

For the reconstruction of the complete genome, reads taxonomically classified as belonging to *Rabies lyssavirus* species were selected and de novo assembled using IDBA-UD v1.1.1 [5] with the multi-kmer approach using a minimum value of 24, a maximum value of 124 and an inner increment of 5. The two contigs produced were blasted online, resulting in >99% similarity with the Genbank record “Rabies virus gene for nucleoprotein, phosphoprotein, matrix protein, glycoprotein complete cds, isolate: BRmk1358” (accession number AB810256.2), which was subsequently used for a reference-based assembly.

High quality reads were aligned against the chosen reference genome using BWA v0.7.12 [6] and standard parameters. Alignments were processed with SAMtools v1.6 [7] to convert them in BAM format e sort them by position. SNPs were called using LoFreq v2.1.2 [8]. According to LoFreq usage recommendations, the alignment was first processed with Picard-tools v2.1.0 (http://broadinstitute.github.io/picard/) and GATK v3.5 [9-11] in order to correct potential errors, realign reads around indels and recalibrate base quality. LoFreq was then run on fixed alignment with option “--call-indels” to produce a vcf files containing both SNPs and indels. From the final set of variants, indels with a frequency lower than 50% and SNPs with a frequency lower than 25% were discarded. To produce the consensus sequence we changed the reference genome in agreement with the following rules: a) for a position j, if coverage was not enough to reliably call variants we added an "N" base; b) for a position j, if coverage was enough to reliably call variants but no SNP were called, we added reference genome base at position j; c) for a position j, if coverage was enough to reliably call variants and at least one SNP were called, we added the nucleotide using the IUPAC nucleotide code (http://www.bioinformatics.org/sms/iupac.html) in according with bases present. Finally, high quality reads were re-aligned with BWA against the consensus sequence produced; we performed a visual inspection of the alignment with tablet v1.14.10.21 [12] and, if required, manually revised the consensus sequence based on this alignment. Moreover, data reporting the entire sequences from the Kinkajou (*Potos flavus*) sample were deposited at the Brazilian System of the National Genetic Patrimony and The Associated Common Traditional Knowledge (https://sisgen.gov.br/paginas/login.aspx), under accession number A4771EF.

**Phylogenetic analysis**

We created two datasets. The first dataset consisted of the complete genome of the RABV from *Potos flavus* from Brazil obtained in this study*,* as well as all the complete genomes (88 sequences) of rabies viruses from the Americas available in GenBank on 18 March 2019, and the N gene of the virus identified in a *Potos flavus* in Peru (JX648546). Three complete genomes from other lyssaviruses were used as an outgroup (KU244267, EF157977, EF157976). The second dataset contained sequences of the N gene representing the distinct genetic groups described by [13], along with the N sequences from bovine and equine samples generated in this study and their corresponding BLAST results.

Sequences were aligned using MAFFT v. 7 [14]. For the complete genome dataset, all the non-coding hypervariable regions were removed. For each dataset, we inferred a maximum likelihood (ML) phylogenetic tree using IQ-Tree v1.6.9 [15] and the best-fit model of nucleotide substitution determined with ModelFinder [16]. One thousand ultrafast bootstrap replicates were performed to assess the robustness of individual nodes of the phylogeny. Phylogenetic trees were visualized with the program FigTree v1.4.2 (<http://tree.bio.ed.ac.uk/software/figtree/>).

**A**

**B**

**Appendix Figure 1.** Detailed analysis of samples collected from the Mato Grosso state during the period 2007 to 2011. **Panel A.** Maximum-likelihood phylogenetic tree of the 183 rabies virus partial Nucleoprotein sequences (603bp). **Panel B.** Geographical distribution of the cases.

Samples collected from bovines (n=162) and equines (n=20) fell within four of the distinct lineages, previously described by Torres and co-authors (2014) [13]. Most of the characterized sequences (140/183) belonged to the group VIb (blue) and were identified in different areas of the Mato Grosso state. This variant has been widely circulating in northeastern Argentina since 1996. Similarly, group Ib (grey), one of the most widespread vampire-bat genetic groups identified in South America (Argentina, Uruguay, Peru and Brazil), encompassed 21/183 viruses detected in different areas of the state. The remaining viruses cluster within groups Id (1 sequence/purple) and IVd (20 sequences/yellow), which were sampled only from the eastern part of the state. To date, both these groups have been identified in Brazil only.

**Host identification**

Nucleic acids extracted trough the QIAamp Viral RNA mini kit (Qiagen) were used as source of host DNA, as no Dnase is used in the procedure. Partial cytochrome oxidase I (COI) -658 bp- was amplified using published primers [17] and the obtained sequence was used for host identification using BOLD (The Barcode of Life Data System) analysis [18]. As no match could be obtained through BOLD, we used the BLAST platform to identify the closest species [2] and we then performed a phylogenetic analysis to solve the taxonomy of the host of the divergent RABV found in the repository. The dataset used included the closest match, identified as the *Potos flavus,* and three sequences for other selected mammals with known rabies cycles in the Americas, all retrieved from GenBank public database. The tree used has been obtained using PhyML [19] and was graphically adapted using Figtree v1.4.2 (<http://tree.bio.ed.ac.uk/software/figtree/>) and Adobe Illustrator. As already shown by the lack of match using the BOLD system, phylogenetic analyses using COI showed great genetic variability between known kinkajous and our sequence (AF 2 panel A). Thus, partial cytochrome B (CYTB) was further amplified [20]. Alignment and phylogenetic analyses were performed as for the COI sequence. However, in this case our sequence was compared with published haplotypes obtained throughout the geographical range of kinkajou, confirming that our host belongs to the species *P. flavus,* subclade 5b, previously reported from the Atlantic forest [21] (AF 2 panel B). A sequence from the coati (*Nasua nasua*) was included in this analysis as outgroup.

A

B

**Appendix Figure 2.** Host identification of BRKJ sample. **Panel A.** Maximum-likelihood phylogenetic tree of a total of 31 partial cytochrome oxidase I (COI) sequences (658 bp), representatives for ten host species among bats, primates and carnivores, known to have a role in rabies maintenance in the Americas. **Panel B.** Maximum-likelihood phylogenetic tree of 19 partial CYTB sequences (697 bp), representatives for different clades and subclades as previously identified by Nascimento et al (2017). CYTB partial sequence from coati (*Nasua nasua*) was included as outgroup.

**REFERENCES**

1. De Benedictis P, De Battisti C, Dacheux L, et al. Lyssavirus detection and typing using pyrosequencing. J Clin Microbiol. 2011;49(5):1932-8.
2. Altschul SF, Gish W, Miller W, et al. Basic local alignment search tool. J Mol Biol 1990;215:403–10.
3. Buchfink B, Xie C, Huson DH. Fast and sensitive protein alignment using DIAMOND. Nat Methods. 2014;12:59– 60.
4. Huson DH, Beier S, Flade I, et al. MEGAN Community Edition - Interactive Exploration and Analysis of Large-Scale Microbiome Sequencing Data. PLoS Comput Biol. 2016;12:1–12.
5. Peng Y, Leung HCM, Yiu SM, et al. IDBA-UD: a de novo assembler for single-cell and metagenomic sequencing data with highly uneven depth. Bioinformatics 2012;28:1420–8.
6. Li H, Durbin R. Fast and accurate long-read alignment with Burrows-Wheeler transform. Bioinformatics. 2010;26:589-95.
7. Li H, Handsaker B, Wysoker A, et al. The Sequence alignment/map (SAM) format and SAMtools. Bioinformatics. 2009;25:2078-9.
8. Wilm A, Aw PP, Bertrand D, al. LoFreq: A sequence-quality aware, ultra-sensitive variant caller for uncovering cell-population heterogeneity from high-throughput sequencing datasets. Nucleic Acids Res. 2012;40(22):11189-201.
9. McKenna A, Hanna M, Banks E, et al. The Genome Analysis Toolkit: a MapReduce framework for analyzing next-generation DNA sequencing data. Genome Research. 2010;20:1297-303.
10. DePristo M, Banks E, Poplin R, et al. A framework for variation discovery and genotyping using next-generation DNA sequencing data. Nature Genetics. 2011;43:491-8.
11. Van der Auwera GA, Carneiro M, Hartl C, et al. From FastQ Data to High-Confidence Variant Calls: The Genome Analysis Toolkit Best Practices Pipeline. Curr Protoc Bioinformatics. 2013;43:11.10.1-33.
12. Milne I, Bayer M, Cardle L, et al. Tablet-next generation sequence assembly visualization. Bioinformatics. 2010;26:401–2.
13. Torres C, Lema C, Dohmen FG, et al. Phylodynamics of vampire bat-transmitted rabies in Argentina. Mol Ecol. 2014;23(9):2340-52.
14. Katoh K, Standley DM. MAFFT multiple sequence alignment software version 7: improvements in performance and usability. Mol Biol Evol. 2013;30(4):772-80.
15. Nguyen LT, Schmidt HA, von Haeseler A, et al. IQ-TREE: a fast and effective stochastic algorithm for estimating Maximum-Likelihood phylogenies. Mol Biol Evol. 2015;32(1):268–74.
16. Kalyaanamoorthy S, Minh BQ, Wong TKF, et al. ModelFinder: fast model selection for accurate phylogenetic estimates. Nat Meth. 2017;14(6):587–9.
17. Folmer O, Black M, Hoeh W, et al. DNA primers for amplification of mitochondrial cytochrome c oxidase subunit I from diverse metazoan invertebrates. Mol Mar Biol Biotech. 1994;3(5):294–299.
18. Ratnasingham S, Hebert PDN. BARCODING, BOLD:The Barcode of Life Data System (www.barcodinglife.org). Mol Ecol Notes. 2007;7:355–364.
19. Dereeper A, Guignon V, Blanc G, et al. Phylogeny.fr: robust phylogenetic analysis for the non-specialist. Nucleic Acids Res. 2008;36:465–469.
20. Irwin DM, Kocher TD, Wilson AC. Evolution of the cytochrome b gene of mammals. J Mol Evol, 1991;32(2):128–144.
21. Nascimento FF, Oliveira-Silva M, Veron G, et al. The Evolutionary History and Genetic Diversity of Kinkajous, *Potos flavus* (Carnivora, Procyonidae). J Mamm Evol. 2017;24(4):439–451.
